# Adhesins of *Brucella*: Their Roles in the Interaction with the Host

**DOI:** 10.3390/pathogens9110942

**Published:** 2020-11-12

**Authors:** Magalí G. Bialer, Gabriela Sycz, Florencia Muñoz González, Mariana C. Ferrero, Pablo C. Baldi, Angeles Zorreguieta

**Affiliations:** 1 Fundación Instituto Leloir (FIL), IIBBA (CONICET-FIL), Buenos Aires 1405, Argentina; mbialer@leloir.org.ar (M.G.B.); gabrielasycz@gmail.com (G.S.); 2 Cátedra de Inmunología, Facultad de Farmacia y Bioquímica, Universidad de Buenos Aires, Buenos Aires 1113, Argentina; fmgonzalez@ffyb.uba.ar (F.M.G.); ferrerom@ffyb.uba.ar (M.C.F.); 3 Instituto de Estudios de la Inmunidad Humoral (IDEHU), CONICET-Universidad de Buenos Aires, Buenos Aires 1113, Argentina; 4 Departamento de Química Biológica, Facultad de Ciencias Exactas y Naturales, Universidad de Buenos Aires, Buenos Aires 1428, Argentina

**Keywords:** *Brucella*, adhesins, Ig-like domain, monomeric autotransporters, trimeric autotransporters, extracellular matrix, polar localization, virulence factors, vaccine candidates, fibronectin

## Abstract

A central aspect of *Brucella* pathogenicity is its ability to invade, survive, and replicate in diverse phagocytic and non-phagocytic cell types, leading to chronic infections and chronic inflammatory phenomena. Adhesion to the target cell is a critical first step in the invasion process. Several *Brucella* adhesins have been shown to mediate adhesion to cells, extracellular matrix components (ECM), or both. These include the sialic acid-binding proteins SP29 and SP41 (binding to erythrocytes and epithelial cells, respectively), the BigA and BigB proteins that contain an Ig-like domain (binding to cell adhesion molecules in epithelial cells), the monomeric autotransporters BmaA, BmaB, and BmaC (binding to ECM components, epithelial cells, osteoblasts, synoviocytes, and trophoblasts), the trimeric autotransporters BtaE and BtaF (binding to ECM components and epithelial cells) and Bp26 (binding to ECM components). An in vivo role has also been shown for the trimeric autotransporters, as deletion mutants display decreased colonization after oral and/or respiratory infection in mice, and it has also been suggested for BigA and BigB. Several adhesins have shown unipolar localization, suggesting that *Brucella* would express an adhesive pole. Adhesin-based vaccines may be useful to prevent brucellosis, as intranasal immunization in mice with BtaF conferred high levels of protection against oral challenge with *B. suis*.

## 1. Introduction

*Brucella* spp. are Gram-negative bacteria that infect several animal species and can be transmitted to humans by several routes, producing one of the most common zoonotic diseases worldwide. A central aspect of *Brucella* pathogenicity is its ability to invade, survive, and replicate in several phagocytic and non-phagocytic cell types, leading not only to chronic infections but also to chronic inflammatory phenomena in different tissues. For both phagocytic and non-phagocytic cells, the first step of the invasion process involves interactions between surface molecular factors of *Brucella* and the host cell, leading to the cellular adhesion of the pathogen. Several *Brucella* proteins have been shown to be involved in the adhesion of this bacterium to different cell types and/or to extracellular matrix (ECM) components. In this review, we describe the main characteristics of these *Brucella* adhesins in the broader context of bacterial adhesins, and how they contribute to the cellular infectious process of brucellae.

## 2. *Brucella* Infection and Clinical Manifestations

With more than 500,000 new cases annually, human brucellosis continues to be one of the commonest zoonotic diseases worldwide. Although this disease has been eradicated in some developed countries, it still constitutes a public health problem in Latin America, the Middle East, North and East Africa, and South and Central Asia [1]. Moreover, the disease is still present in some European countries.

*Brucella* spp. are Gram-negative non-capsulated and non-sporulated bacilli or cocobacilli that lack cilia or flagella. Despite the great number of *Brucella* species identified, which may infect domestic animals and wild animals, only *B. melitensis* (goats and sheep), *B. suis* (pigs), *B. abortus* (cattle), and, to a minor extent, *B. canis* (dogs) are linked to human brucellosis. Different *Brucella* species yield smooth (S) or rough (R) colonies when cultured in agar, which is a difference that is directly related to the structure of the lipopolysaccharide (LPS). The LPS is divided into three regions: the lipid A (the innermost portion), a polysaccharidic core, and the O polysaccharide (the outermost portion). Smooth species (*B. melitensis*, *B. suis*, *B. abortus*, and others) produce a “complete” LPS (S-LPS) containing the three portions, whereas rough species (*B. canis* and *B. ovis*) produce a rough LPS (R-LPS) that lacks the O polysaccharide.

*Brucella* infection in humans is mainly acquired through the consumption of raw animal products, inhalation of contaminated aerosols in slaughterhouses, rural settings or laboratories [2], and contact of the abraded skin with contaminated tissues or materials. Less frequently, accidental infection with attenuated vaccine strains [3,4,5,6] and vertical transmission have been reported [7].

Human brucellosis has a wide spectrum of clinical manifestations, which depend on the stage of the disease and the organs and systems involved. The disease usually presents as a febrile illness accompanied by myalgia, arthralgia, and hepatomegaly, and may evolve with an uncomplicated course or may present complications involving particular organs or systems [8]. Osteoarticular involvement is the most common focal complication [9]. The diversity of tissues that can be affected by *Brucella* is likely related to its ability to invade, survive, and replicate in several phagocytic and non-phagocytic cell types, as explained below. Despite its tendency to produce chronic illness and even disabling disease, human brucellosis is only rarely fatal. In animals, the most prevalent manifestations are abortions, reduced fertility, weight loss, and reduced milk production [10]. 

## 3. *Brucella* Entry into Host Cells

A central aspect of *Brucella* pathogenicity is its ability to invade, survive, and replicate in several cell types, leading not only to chronic infections but also to chronic inflammatory phenomena that explain most of the clinical manifestations of brucellosis [11]. Most initial research was performed using murine macrophagic cell lines [12], bovine macrophages [13], human monocytes [14], and widely used non-phagocytic cell lines such as HeLa (human cervical cells) or Vero (kidney, African green monkey) [15,16]. However, further in vitro studies revealed that *Brucella* is capable to infect and replicate in human osteoblasts [17], synoviocytes [18], trophoblasts [19], endothelial cells [20], lung epithelial cells [21], dendritic cells [22], and hepatocytes [23] as well as in murine alveolar macrophages [24], canine trophoblasts and phagocytes [25], and ovine testis cells lines [26]. 

The internalization of *Brucella* into the different cell types is a complex multi-stage process. Whatever the host cell (phagocytic or non-phagocytic) and the *Brucella* strain involved (smooth or rough), the first step in this process involves interactions between surface molecular factors of both the host cell and the pathogen leading to cellular binding of the bacterium. In fact, it was shown by scanning electron microscopy that *B. abortus* adheres (as early as 1 h after infection) and forms bacterial aggregates on the surface of host cells in a time-dependent manner [27]. Whereas several of the surface molecular factors involved have been identified, the full repertoire of molecular components and mechanisms acting on either active bacterial penetration or passive uptake of *Brucella* spp. are not fully characterized [28]. For non-opsonized bacteria, internalization into macrophages seems to depend on lipid rafts present in the plasma membrane of these cells [29,30]. It has been shown that lipid rafts-associated molecules, including cholesterol and the ganglioside GM1, are involved in the entry of *B. suis* into murine macrophages under non-opsonic conditions [31]. In addition, a class A scavenger receptor (SR-A) seems to be required for *B. abortus* internalization into macrophages through a lipid raft-mediated mechanism [32]. These three host molecules have been involved in the ability of naturally rough *Brucella* species (*B. ovis*, *B. canis*) to infect murine macrophages [33]. Although these lipid raft-associated molecules have a role in *Brucella* internalization in macrophages, it has not been determined whether they participate in bacterial adhesion or, alternatively, only contribute to bacterial penetration or uptake.

In addition to these lipid raft-associated molecules, other host components have been identified as being involved in the interaction between brucellae and the host cells. Sialic acid-containing molecules were proposed to be involved in the interaction of brucellae with macrophages and epithelial cells [27]. GM1 is a sialylated molecule, which may perhaps explain its role in lipid raft-mediated internalization in macrophages. This study also produced evidence suggesting that cell surface heparan sulphate molecules may be involved in *Brucella* binding to epithelial cells. Based on the hypothesis that the interactions of *Brucella* with the ECM contribute to the spread of the bacteria through tissue barriers, the ability of the pathogen to bind to ECM constituents was also explored. It was shown that *B. abortus* binds in a dose-dependent manner to immobilized fibronectin and vitronectin and, to a lesser extent, to chondroitin sulphate, collagen, and laminin [27]. 

As mentioned above, adhesion to host cells is the first step of the infectious cycle of many pathogens. Most bacterial pathogens express adhesins and other molecules that mediate the binding to a wide range of cell surface molecules and ECM components depending on the lifestyle of the microorganism. The fact that *Brucella* species can bind to the cell surface and ECM components strongly suggests the expression of bacterial molecules involved in such an interaction. Although not formally shown to be involved in adhesion, *Brucella* LPS has been linked to the internalization of the pathogen in macrophages. Smooth *B. abortus* strains expressing a complete LPS (including the O-polysaccharide) enter macrophages through lipid-rafts, whereas a rough mutant does not [30,34]. However, naturally rough *Brucella* species (*B. ovis, B. canis*) seem to use lipid rafts for entry [33], suggesting that lipid raft-mediated internalization of brucellae does not depend on O-polysaccharide expression. A role for some outer membrane proteins, namely Omp22 and Omp25, in *Brucella* binding or internalization has also been suggested. Targeted inactivation of their corresponding genes impaired internalization of rough *B. ovis* but not that of *B. abortus* [35,36]. Moreover, *B. abortus* mutants were more adherent than the wild-type strain. While the role of the LPS and outer membrane proteins in the ability of *Brucella* to adhere to cells or ECM requires further clarification, more recent studies have led to the identification of bacterial adhesins clearly involved in these adhesion processes (see below).

Upon entry into the host cells, *Brucella* organisms initiate an intracellular cycle that involves a sequential traffic through the endocytic, secretory, and autophagic compartments. Bacterial effectors delivered inside the infected cells through a type IV secretion system encoded by the *virB* operon are essential to accomplish these steps [15,37,38,39]. The O polysaccharide of the LPS is also involved in the ability of *Brucella* to establish intracellular infections. Phagosomes containing smooth strains of *B. suis* do not fuse with lysosomes, at least in murine macrophages, whereas those harboring rough mutants rapidly fuse [40]. This seems to be related to the fact that only the naturally smooth strains enter the cells through lipid-rafts and can inhibit phagosome-lysosome fusion [30,34].

## 4. Bacterial Adhesins

Most pathogenic bacteria interact with their hosts through adhesive molecules (adhesins) that are exposed on their cell surfaces. Since adhesion to host cells can also stimulate immune activation, several bacteria produce a surface layer (i.e., capsular polysaccharide) that prevents immune recognition or phagocytosis. For this reason, they often express adhesins on polymeric structures that extend out from the cell surface at a prudential distance. For some bacteria, attachment to the host cell surface is also crucial for effector injection through complex secretion systems. Lastly, adhesion to the host cell is the previous step to internalization for those bacteria whose strategy to achieve proliferation and survival is the intracellular life [41,42]. The adhesins can be grouped into two types: (1) filamentous (fimbrial) adhesins consisting of complex structures made up of multiple subunits and (2) non-fimbrial adhesins that can be monomeric or trimeric proteins. 

Fimbrial adhesins are a varied group of polymeric fibers that are visible using electron microscopy. In Gram-negative bacteria, these adhesins can be classified into: (1) the chaperone-usher pili (CUP), (2) the alternative chaperone-usher pathway pili, (3) Type IV pili, and (4) pili assembled by the extracellular nucleation-precipitation pathway (curli) [41,43]. The subunit at the tip of the CUP pili is a lectin that can bind sugar-containing molecules on the host cell surfaces [44]. The Type IV pili are long filaments composed of pilin subunits assembled into bundles, which are involved in diverse functions including bacterial twitching motility, auto-aggregation, and attachment to host cells [45]. Curli are involved in many physiological and pathogenic processes such as biofilm formation and host cell adhesion and invasion. The curli are assembled via the nucleation-precipitation pathway and display structural similarities with functional amyloids [46,47]. As mentioned below, *Brucella* spp. do not seem to express fimbrial adhesins. 

Non-fimbrial adhesins include adhesins that belong to the RTX (repeat in toxin) protein family and those that correspond to type V secretion systems (T5SS), which are also called autotransporter proteins. 

RTX adhesins are secreted by a type 1 secretion system (T1SS) that has three components: an inner-membrane ABC (ATP binding cassette) transporter, a membrane fusion protein, and an outer-membrane pore from the TolC family. The substrates of T1SSs do not harbor an N-terminal cleavable signal peptide but share a structural C-terminal domain that is not cleaved off during the secretion process [48]. The RTX adhesins are usually loosely attached to the bacterial surface and have been implicated in bacteria-to-bacteria interactions during biofilm formation and adhesion to epithelial cells [49].

The T5SSs (subfamilies Va–Ve) play important roles in the interaction of several pathogens with their hosts [50]. Originally, the term “autotransporter” was proposed because it was thought that all the information for its translocation from the inner membrane to the extracellular medium was mostly contained in the protein itself. This concept has changed since other factors, such as chaperones and the BAM (β-barrel Assembly Machinery) system are required for secretion of these proteins. Furthermore, more recently, it was shown that another system, the TAM (Translocation and Assembly Module) complex, is also required for the correct translocation of autotransporters into the outer membrane [51,52,53]. It was proposed that this complex spanning the periplasmic space might solve the energy problem to translocate proteins through the outer membrane [52]. Therefore, the current model proposes that the TAM and BAM systems would act in a concerted manner [51,54].

The T5SS or autotransporter proteins share common structural and functional characteristics: (1) an N-terminal Sec-dependent signal peptide that mediates the transport from the cytoplasm to the periplasm, (2) a passenger (and functional) domain, and (3) a C-terminal β-barrel domain that forms a pore in the outer membrane through which the passenger domain is translocated to the cell surface [55]. In the subclass Va, the autotransporters are monomeric and the passenger and secretion domains are integrated into the same protein, the β-barrel domain forms a pore of 12 antiparallel β-strands, and the passenger regions consist of highly variable repetitive amino acid motifs. Some of these autotransporters are important virulence factors, playing diverse functions in the interaction with the host. The passenger domains have often enzymatic activity and usually adopt a repetitive β-helix fold extending away from the bacterial cell surface, as demonstrated by the crystal structure of the Pertactin passenger domain [56]. Passengers with enzymatic activity are cleaved off from the surface while adhesion passengers can be retained on the cell surface without cleavage (for a comprehensive review, see Reference [50]). Some adhesins of the monomeric autotransporter family have been described in *Brucella* spp., as explained below.

In the two-partner secretion systems (T5SS type Vb), the passenger and β-translocator domains are encoded by two different genes. Filamentous haemagglutinin adhesins are exported by this type of system. These adhesins are often involved in a tight interaction with a host cell receptor and also in biofilm formation [50]. 

All members of the trimeric autotransporter Type Vc group that have been characterized so far are implicated in adhesion functions. They usually bind to host receptors or to host ECM components. As detailed in the next section, *Brucella* spp. express adhesins that belong to this subclass of autotransporters. While the overall organization of these proteins is similar to that of the monomeric autotransporters, they contain a shorter C-terminal translocation domain of 50–100 amino acids and the 12 β-strand pore is achieved by protein trimerization. Usually, the passenger domain harbors conserved structural elements named as head, connector, and stalk domains. The combinations of these repeats result in either “lollipop structures” like YadA or as “beads-on-a-string” like BadA [52]. Although the head domains typically mediate adhesion to host targets, the stalk domains can also participate in adhesion functions. Internal regions may serve to extend the head domain away from the bacterial cell surface. Unlike several monomeric autotransporters, trimeric autotransporters are not released into the extracellular space [50]. 

The Type Ve of T5SS harbors a 12-stranded β-barrel domain and a secreted, monomeric passenger domain that remains attached after translocation. The main difference with type Va autotransporters is that the type Ve have an inverted domain order with the β-barrel at the N-terminal end and the passenger domain at the C-terminus, and, thus, are named as “inverse autotransporters” [57]. Well-known examples are the intimin and invasin from pathogenic *Escherichia coli* and *Yersinia* spp., respectively. The passenger domains of this type of T5SS contain domains with Immunoglobulin (Ig)-like or lectin-like structures. The intimin of enteropathogenic and enterohemorrhagic *E. coli* strains mediates an intimate contact with the Tir receptor, which is delivered by the bacterium to the surface of the host cell. The invasin of *Yersinia* spp. binds directly to β1-integrins on the apical side of gut epithelial cells, which promotes bacterial internalization via endocytosis [58,59]. 

## 5. Adhesins of *Brucella*

**Figure 1:**
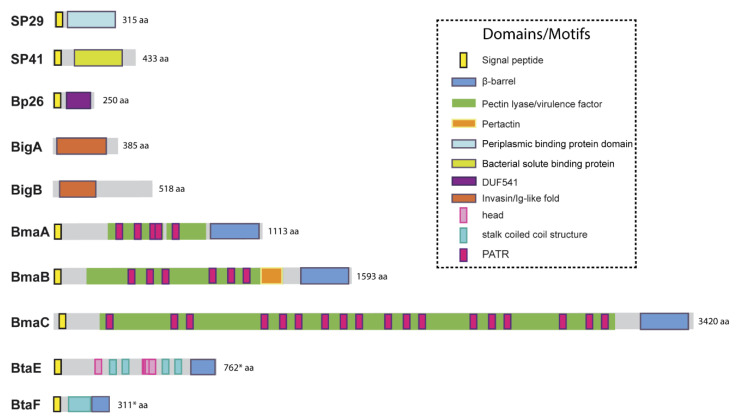
Domain organization of described *Brucella* adhesins. Schematic representation of the adhesins described in *Brucella* spp, showing functional and structural domains predicted by bioinformatics (SignalP 5.0, Pfam, BLAST, InterProScan). Asterisks indicate the cases for which start codons upstream of the annotated ORF were identified and the translation product of the new ORF contained an N-terminal signal peptide with a reliable score. aa: amino acids.

The genomes of *Brucella* spp. do not harbor loci associated with components of pili or curli that could function as fimbrial adhesins. Furthermore, by electron microscopy, no pilus-like structures have been observed. However, several non-fimbrial adhesins have been identified that were shown to have a role in the interaction with the host. A diagram of these adhesins, showing their domains, is depicted in Figure 1, and additional information is presented in Table 1.

### 5.1. Unclassified Adhesins

Due to the abundance of carbohydrates in the surface of red cells, hemagglutination tests have been used for the detection and characterization of many lectin-like adhesins in bacterial pathogens. Using this approach, Rocha-Gracia et al. found that *B. abortus* and *B. melitensis* can agglutinate human (A+ and B+), hamster, and rabbit erythrocytes, and that this activity was associated with a bacterial 29-kDa surface protein (SP29) that binds to these cells [60]. Purified SP29 bound directly to rabbit erythrocytes, and this binding was abolished by neuraminidase treatment of red cells, indicating that SP29 binds to sialic acid-containing receptors. The analysis of an internal fragment obtained by peptic digestion suggested that SP29 is a D-ribose-binding periplasmic protein precursor found in *B. melitensis* (BruAb2_0373) (Figure 1). No further characterization of this protein or its importance for *Brucella* pathogenesis has been reported despite the demonstration that *B. melitensis* is able to invade erythrocytes in vivo at least in the mouse model [72]. This later study revealed that *B. melitensis* can adhere to murine erythrocytes as early as 3-h post-infection but is later found mainly in the cytoplasm of these cells. Moreover, erythrocytes represented the major fraction of infected cells in the bloodstream. Purified erythrocytes from infected mice were able to transmit *B. melitensis* infection to naïve mice. 

**Figure 2:**
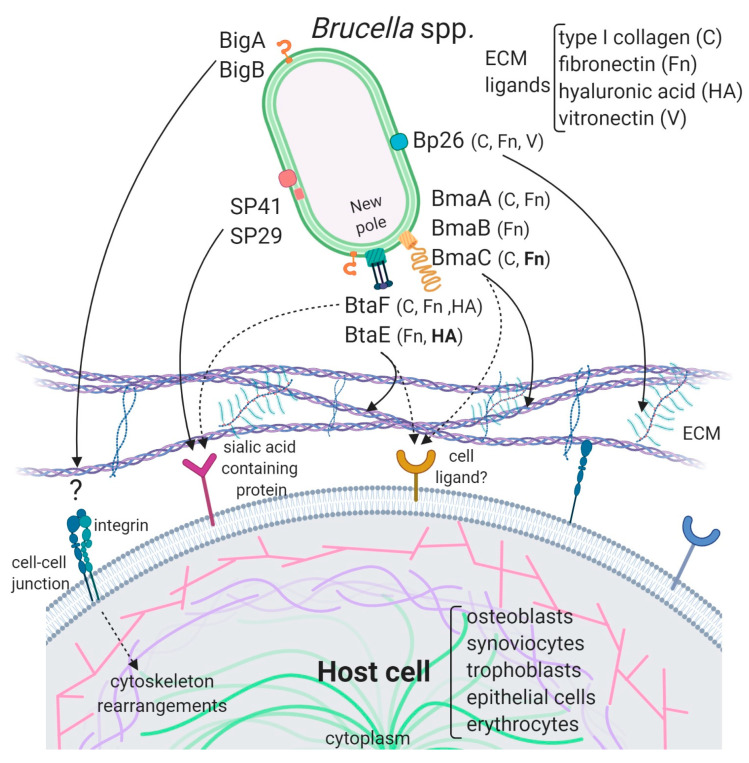
Model of the interaction of *Brucella* adhesins with host cells. The described *Brucella* spp. adhesins are depicted on the bacterial surface and their interactions with ECM components and ligands on the host cell are represented by black arrows. ECM components and cell types to which these adhesins bind are mentioned. ECM ligands in bold are supported by consistent evidence while those in a normal font are supported by indirect evidence. The Bma and Bta proteins are mostly localized at the new pole generated after asymmetric bacterial division. Bipolar localization was shown for BigA, but BigB polarity has not been determined. SP29 is predicted to be periplasmic while SP41 was shown to be exposed on the bacterial surface. It is not clear if Bp26 is localized to the outer membrane or in the periplasm. The cell ligands for Big and Bp26 adhesins have not yet been identified. In addition to ECM, Bma and Bta adhesins could interact with cell surface ligands. Dotted arrows represent putative interactions. Importantly, while all the *Brucella* adhesins characterized to date are shown, they may be not simultaneously expressed on bacteria.

To our best knowledge, the first *Brucella* adhesin for which a functional role was fully characterized in vitro was SP41 (Figure 1, Table 1) [61]. This protein is the predicted product of the *ugpB locus*, which encodes a protein of 433 amino acids with similarity to a periplasmic glycerol-3-phosphate-binding ATP-binding cassette (ABC) transporter protein found in several bacterial species, and harbors a bacterial solute-binding protein domain. Immunofluorescence studies indicated that SP41 is surface exposed, and antibodies directed to SP41 inhibited *B. suis* adherence to HeLa cells. Notably, a Δ*ugpB* *B. suis* mutant exhibited a significant reduction in the adherence to epithelial cells, supporting the contention that SP41 is an adhesin. Treatment of HeLa cells with neuraminidase abolished SP41 binding to these cells, suggesting the involvement of sialic acid residues in this interaction (Figure 2, Table 1). In contrast, a further study in *B. ovis* did not reveal an effect of *ugpB* deletion on early internalization or intracellular survival of this rough species in murine macrophages (J774.A1 cell line) or HeLa cells [62]. In addition, the deletion had no effect on the ability of *B. ovis* to colonize the spleen after intraperitoneal inoculation in mice. The *ugpB* gene seems to be functional in *B. ovis* as revealed by RT-PCR assays, and the encoded protein differs only by five amino acids from that of *B. suis*. It was argued that other adhesins would be more exposed on the bacterial surface of *B. ovis* due to the absence of O-polysaccharide chains, favoring their interaction with the host cell.

The potential role of the *Brucella* Bp26 protein as an adhesin was recently tested in vitro [66]. The rationale exposed by the authors for testing this protein was not related to structural or homology criteria, but to the fact that Bp26 induces strong antibody responses in infected individuals [73]. Bp26 is a 250 amino acid-predicted protein with a domain of unknown function (DUF541) (Figure 1). The binding properties of Bp26 to ECM components such as type I collagen, fibronectin, vitronectin, and laminin were tested by ELISA and biolayer interferometry. According to the results of these assays, Bp26 binds to both immobilized and soluble type I collagen and vitronectin, and to soluble (but not immobilized) fibronectin, but does not bind to laminin (Figure 2, Table 1). The relevance of Bp26 for in vitro adhesion of *Brucella* to cells or for the outcome of in vivo infections has not been tested.

### 5.2. Adhesins Containing Ig-Like Domains

A study by Czibener and Ugalde allowed the identification of a pathogenicity island in *B. abortus* (BAB1_2009-2012) whose deletion resulted in a reduced attachment of the bacterium to HeLa cells [63]. Furthermore, the deletion mutant also displayed a reduced capacity to colonize the spleen of mice after oral infection as compared to the wild-type strain. In particular, BAB1_2009 was found to encode a protein that harbors a bacterial Ig-like (BIg-like) domain present in adhesins from the invasin/intimin family [74] (Figure 1). In the following study, the role of this protein (named as BigA) in adhesion to epithelial cells was demonstrated [64]. This study also revealed that BigA is an exposed outer membrane protein and that incubation of the bacteria with antibodies against the Ig-like domain of BigA before infection of HeLa cells reduces the number of intracellular bacteria. While a deletion mutant strain displayed a significant defect in both adhesion and invasion to polarized epithelial cell lines such as Caco-2 (human colon) and Madin–Darby canine kidney (MDCK), overexpression of the *bigA* gene greatly increased them (Table 1). Confocal microscopy analyses showed that the BigA adhesin targets the bacteria to the cell-cell junction membrane in confluent epithelial cells and also induces cytoskeleton rearrangements (Figure 2). A recent publication by the same research group showed that other Ig-like (Blg-like) domain-containing protein (BAB1_2012, named BigB) (Figure 1), encoded by the same locus (BAB1_2009-2012), is also involved in adhesion to epithelial cells and targets proteins involved in cell-cell and cell-matrix interactions (Figure 2) [65]. The Δ*bigB* mutant showed a significant reduction in intracellular bacteria at the early stages of infection both in HeLa cells and in polarized MDCK cells (Table 1). It was further demonstrated by counting fluorescent bacteria that the phenotype on HeLa cells was due to a defect in adhesion. Similar to BigA, recombinant BigB induced profound cytoskeleton rearrangements in HeLa cells (Figure 2). HeLa cells transfected with focal adhesion markers showed changes in focal adhesion sites. It was proposed that, similar to BigA, BigB targets proteins in cell-cell junctions, which, in turn, triggers changes in the cytoskeleton (Figure 2). This work also showed that the BAB1_2011 gene encodes a periplasmic protein (PalA), which is necessary for the proper display of both the BigA and BigB adhesins, indicating that the genomic island is dedicated to the adhesion of *Brucella* to host cells. Although the phenotypes of the *big* mutants have not been tested in vivo, the previous result obtained with the BAB1_2009-2012 deletion mutant strongly suggests that the Big adhesins have a role in vivo.

### 5.3. Autotransporters

Numerous virulence factors of bacterial pathogens contain domains or motifs related to adhesion to biotic or abiotic surfaces. A comprehensive search for conserved adhesion-associated domains/motifs in the *B. suis* 1330 genome and subsequent phylogenetic analyses revealed the presence of three clearly separated groups of adhesins: (1) monomeric autotransporters (BRA0173, BR2013, BRA1148), (2) trimeric autotransporters (BR0072 and BR1846), and (3) Ig-like domain containing-proteins (BR2009 and BR2012) [75]. Other proteins with no clear associated functions were also identified but not described in this work. Group 2 also included the protein BR0049. We have recently shown that BR0049 is certainly not an adhesin, but is required for the correct insertion of proteins from the autotransporter families (see below) [53]. Group 3 comprised orthologous proteins of the *B. abortus* BigA and BigB proteins described above.

#### 5.3.1. Monomeric Autotransporters

As mentioned above, *B. abortus* binds in a dose-dependent manner to components of the ECM, such as fibronectin and vitronectin [27]. In an attempt to identify *B. suis* genes encoding proteins that might be involved in the binding of brucellae to fibronectin, Posadas et al. [67] panned an M13 phage display library of the *B. suis* 1330 genome against immobilized fibronectin. Several recombinant phages showed affinity to immobilized fibronectin. However, only one expressed a portion of a protein that was predicted to be exposed on the cell surface. This protein corresponded to one of the monomeric autotransporters described above (BRA1148) and was named as BmaC for *Bucella* monomeric autotransporter. This protein exhibits all the characteristics of this protein family. BmaC is a large 340 kDa-protein with a long N-terminal cleavable 72 amino acid signal peptide and several adhesion-related motifs within the passenger domain, an extended pectin lyase virulence factor domain, and several passenger-associated-transport-repeats (PATR) (Figure 1). The portion of BmaC expressed on the phage also exhibits affinity (although much less) for type I collagen, suggesting that this very large protein might contribute to the interaction of *B. suis* with other ECM ligands.

Several lines of evidence have shown that BmaC of *B. suis* 1330 mediates the binding of *B. suis* to epithelial cells through cellular fibronectin. The attachment of *B. suis* to HeLa cells was inhibited by the φ-BRA1148 recombinant phage in a dose-dependent manner. A *bmaC* deletion mutant was impaired in the attachment to immobilized fibronectin and to the surface of HeLa and A549 epithelial cells (Table 1). Furthermore, the *bmaC* defective mutant was outcompeted by the wild-type strain in co-infection experiments, and anti-BmaC and anti-fibronectin antibodies significantly inhibited the binding of *B. suis* to HeLa cells [67]. Immunofluorescence microscopy showed that all bacteria with a detectable fluorescent signal displayed BmaC at only one pole, indicating that BmaC is polarly exposed on the cell surface (Figure 2). This is not surprising since several monomeric autotransporters are exposed on the bacterial surface at one pole [76,77]. Confocal microscopy analysis showed the presence of some small GFP-labelled bacterial aggregates on the surface of HeLa cells, mostly in cell boundaries. Single bacteria were found interacting through one of their poles with the cell surface on both the cell body and cell protrusion. Occasionally, polar BmaC was located at the pole interacting with the cell. These observations suggested that the polar localization of BmaC could be relevant in the interaction with host cells in vivo [67].

The monomeric autotransporter proteins encoded by BR0173 and BR2013 (BmaA and BmaB, respectively) of *B. suis* 1330, although much smaller, share significant sequence similarities with BmaC (Figure 1). It was reported that a mutant of *B. suis* 1330, deficient in BmaB (previously called OmaA), is cleared from spleens of BALB/c mice faster than the wild-type strain (between the third and the ninth week post infection), suggesting that BmaB is required during the chronic phase of infection [78]. A recent study [68] indicated that the *bmaB* locus from all *B. abortus* strains analyzed and both the *bmaA* and *bmaC* loci from all *B. melitensis* strains seem to correspond to pseudogenes, while, in *B. suis*, all the Bma proteins could be functional in several strains of this species. In line with these observations, gain or loss of function studies indicated that, at least in *B. suis* strain 1330, BmaA, BmaB, and BmaC proteins contribute, to a greater or lesser degree, to bacterial adhesion among different cell types, such as epithelial (HT-29 and Caco2), synoviocytes, osteoblasts, and trophoblasts (Table 1, Figure 2). These observations show that there are variations in the repertoire of functional adhesins in *Brucella* spp. and open the possibility that these adhesins are involved in host preferences. BmaB was also found at the new pole generated after cell division [68]. 

#### 5.3.2. Trimeric Autotransporters

As mentioned above, the search for conserved adhesion-associated domains/motifs in *B. suis* 1330 identified a group of trimeric autotransporters, including BR0072 and BR1846, which were named BtaE and BtaF, respectively. The *B. suis* BtaE trimeric autotransporter is a 740 amino acid protein that harbors several regions corresponding to the head and the neck subdomains in addition to a connector region and the β-barrel translocator domain (Figure 1). Different genetic approaches showed that BtaE of *B. suis* is involved in the adhesion to ECM components and host cells [69]. The BtaE-defective strain exhibited a decreased ability to adhere to HeLa and A549 epithelial cells and was outcompeted by the wild-type *B. suis* strain for the adhesion to HeLa cells (Table 1). Expression of BtaE in a “non-adherent” *E. coli* strain increased the binding of this heterologous bacterium to immobilized hyaluronic acid and fibronectin. On the other hand, *btaE* deletion impaired bacterial adhesion to hyaluronic acid but had no effect in the adhesion to fibronectin, suggesting that other fibronectin-binding adhesins (such as BmaC) could compensate somehow for the absence of BtaE. The adhesion of the wild-type strain to HeLa cells decreased in the presence of hyaluronic acid, while this compound had almost no effect in the attachment of the *btaE* mutant to these cells, supporting the hypothesis that BtaE mediates the binding of *Brucella* to hyaluronic acid. Therefore, BtaE could also participate in *Brucella* dissemination to different target tissues such as cartilage, heart, and bone, which may result in brucellosis complications. In vivo experiments using the mouse model indicate that the BtaE adhesin is necessary for a successful infection. In effect, a significantly lower number of bacteria were recovered from spleens of animals inoculated through the intragastric route with the *btaE* mutant compared to those inoculated with the wild-type strain [69].

In a subsequent work, it was shown that the BtaE orthologue of *B. abortus* 2308 is also involved in adhesion to epithelial cells. Compared to *B. suis* BtaE, the ortologue of *B. abortus* is much larger and contains a higher number of repetitive adhesion motifs. Furthermore, the *btaE* gene of *B. suis* and *B. abortus* are under the regulation of different mechanisms (see below) [79]. The *btaE* deletion mutant of *B. abortus* 2308 showed a significant reduction in the adhesion to HeLa cells when compared with the wild-type strain, demonstrating that the BtaE variant of *B. abortus* 2308 contributes to the interaction of *Brucella* with the host cell surface to a similar extent to that observed for the *B. suis* 1330 orthologue [69]. 

The regulation of *btaE* at a promoter level was analyzed by Sieira et al. [79]. Comparison of *btaE* promoter sequences among different *Brucella* species revealed that a novel HutC binding site in the promoter region of *btaE* from *B. abortus* 2308 was generated de novo recently in the evolution of the genus. HutC, which is a regulator of the histidine metabolism, also acts as a co-activator contributing to modulation of expression of the *Brucella virB* operon [80]. Moreover, additional transcriptional factors (MdrA and IHF) binding sites were identified in the *btaE* promoter of *B. abortus*. The target-DNA sequences were confirmed by EMSA and DNAseI footprinting assays. 

The HutC binding site is not present in the *btaE* promoters of other *Brucella* strains since it is interrupted by a cytosine. In effect, an electrophoretic mobility shift assay showed that HutC is not able to interact with the *btaE* promoter of *B. suis* 1330, even though the IHF and MdrA showed a binding pattern similar to that observed for the *btaE* promoter of *B. abortus* 2308. Based on these findings, it was proposed that, as a result of the cis regulatory gain of function, the *btaE* promoter acquired the ability to fine-tune its transcriptional output in response to changes in environmental parameters such as nutrient availability. Thus, differential *btaE* expression might generate phenotypic diversity at the regulatory level of adhesins, which might contribute to reciprocal selection between *Brucella* species and their mammalian hosts.

The BtaF trimeric autotransporter of *B. suis* 1330, encoded by the BR1846 annotated locus, is a small protein that harbors an N-terminal peptide signal, a 170 amino acid passenger domain, and a YadA-like C-terminal translocator region (β-barrel translocator domain) (Figure 1). Unlike BtaE, the BtaF protein does not show the presence of conserved adhesion motifs. Analysis with the TA Domain Annotation (daTAA) server [81] showed that most of the passenger domains correspond to a coiled coil stalk but none associated with the “head” structural region [70]. In addition, a careful analysis of the annotated *btaF* upstream region indicated that the ORF starts earlier, adding 33 additional amino acids at the N-terminal sequence. Using a new version of the program to predict the structural features of trimeric autotransporters and the alternative ORF, it was possible to identify a region at the N-terminus that would correspond to the head, even though the structure would be different from those described so far [82]. 

BtaF of *B. suis* 1330 has shown to be promiscuous in its ability to bind to different substrates. The heterologous expression of this small trimeric protein markedly increased the adhesion of non-adherent *E. coli* to HeLa cells and various substrates such as fibronectin, fetuin (a sialic acid-rich protein), hyaluronic acid, and collagen I and also to an abiotic surface such as polystyrene [70] (Table 1, Figure 2). In agreement with these observations, the *btaF* deletion mutant of *B. suis* showed a significant reduction in the ability to bind to fetuin, hyaluronic acid, and collagen I, and in the adhesion to an abiotic surface, even though the adhesion to fibronectin was not affected. Again, as it was observed for the *btaE* mutant, overlapping functions with other adhesins (such as Bma proteins and BtaE) may account for the lack of a Δ*btaF* phenotype toward fibronectin. The BtaF-defective strain showed a significant reduction in the attachment to HeLa and A549 epithelial cells. 

Notably, BtaF was required for complete virulence in mice infected through the oral route (intragastric administration) [70]. The strain lacking BtaF showed a reduction of about one log in the number of bacteria recovered from spleen at early stages of infection. The absence of both trimeric adhesins (BtaE and BtaF) resulted in a more severe phenotype in vivo compared with the attenuation observed for the single mutants. It is possible that some of the functions might be shared or complementary between BtaE and BtaF, while others could be exclusive to BtaF or BtaE. An indirect ELISA assay on sera from healthy and sick pigs infected with *B. suis* suggested that both adhesins are expressed in vivo in the natural host (swine), supporting the role of these adhesins in the infection process. Recently, it was shown that BtaF is also required for virulence in mice after inoculation via a respiratory (intratracheal administration) route [71]. In this case, the splenic load of the deletion mutant was significantly reduced at 7-days and 30-days post-infection as compared to the wild-type strain.

Smooth *Brucella* strains prevent detection by complement partly due to a distinctive structure of its LPS [83,84]. However, it was proposed that, in addition to the LPS, other surface factors mediate the varied sensitivity of *Brucella* species to the bactericidal action of serum [84]. In addition, it has been shown that, in contrast to human serum, components present in murine normal serum do not opsonize smooth *B. abortus* [85]. The *btaF* mutant showed a significantly reduced survival in the presence of 50% porcine serum compared with the wild-type strain [70]. Furthermore, an *E. coli* strain expressing BtaF showed a more than ten-fold increase in the survival percentage in 8% porcine serum as compared with the control strain. Both strains showed similar levels of survival in heat-inactivated porcine serum, suggesting that BtaF is involved in the resistance to complement-mediated serum killing. 

Similar to BmaC and BmaB, BtaE and BtaF adhesins were found to be polarly localized on the bacterial surface [69,70] (Figure 2). Again, the trimeric adhesins were detected in a low proportion of bacteria but, in all cases, the signal showed unipolar localization and, in some cases, sub-polar localization. As it was observed for BmaC and BmaB, they were found at the same pole as AidB-YFP (a new pole marker) [86] and at the opposite of the PdhS-eGFP labeling (old pole marker) [87]. It was proposed that the new pole generated after the asymmetric division would be functionally differentiated for adhesion. An attractive hypothesis is that the initial adhesion of *Brucella* to the host cell would be mediated by adhesins located at the new pole and that adhesin expression only occurs in an infectious bacterial subpopulation. Various cellular mechanisms such as asymmetric division, polar growth, and polar functions generate two functionally differentiated cells [88,89]. Polar localization could be a way of increasing the adhesive power by concentrating the adhesins in a particular region. In fact, host invasion by a bacterial pole can facilitate entry because of the bacterial shape [90]. It is important to note that polar adherence to surfaces is a conserved mechanism shared by several *Alphaproteobacteria* [91,92].

#### 5.3.3. Autotransporters Insertion in the Outer Membrane 

Autotransporter translocation into the outer membrane is assisted by the BAM machinery and associated chaperones. More recently, it was shown that the TAM system, made up of TamA and TamB, is also required for the correct insertion of autotransporters from the *Gammaproteobacteria* group (see above) [93]. 

As mentioned, during the construction of the phylogenetic tree, the BR0049 protein came out as a possible adhesin from the autotransporter family likely due to some structural similarities with this family of proteins. However, our in silico analysis and other reports [94] indicated that BR0049 and its orthologues from other *Alphaproteobacteria* are phylogenetically related to members of the TamB family from *Gammaproteobacteria*. TamB is a large protein mostly periplasmic but inserted in the inner membrane through a non-cleavable signal peptide. BR0049 of *Brucella* spp. shares a relatively low identity (around 22%) with TamB from *Gammaproteobacteria*, but, similarly to this protein, it contains a membrane anchor signal at the N-terminus, which is followed by a region with an abundant β-helix structure, and, at the C-terminus, a short β-barrel structure within the conserved DUF490 domain. As it was proposed for TamB from *Gammaproteobacteria*, it was demonstrated that BR0049 is required for the correct insertion in the OM of the *B. suis* BmaB monomeric autotransporter. In addition, BR0049 was required for complete virulence in mice infected through the intragastric route [53]. The BR0049 mutant showed an increased sensitivity to polymyxin B, lysozyme, and Triton X-100, and, thus, BR0049 was named as MapB (Membrane altering protein). Several results indicated that MapB of *Brucella* plays functions that go beyond that of assisting in autotransporter assembly, suggesting that the TAM machinery would be involved in cell envelope biogenesis.

### 5.4. Brucella Adhesins as Vaccine Candidates

Vaccination is a key health measure in the control and prevention of infectious diseases. In the case of brucellosis, it is necessary to control bacterial dissemination by vaccination of natural hosts as well as vaccination of people professionally exposed to *Brucella* spp. infection. Commercially available *Brucella* vaccines approved for use in animals are based on attenuated strains. These vaccines have serious disadvantages such as producing abortion in pregnant females, being virulent for humans [95,96,97], and inducing immune responses that interfere with animal serological diagnosis [98]. Thus, there is a need to develop safer and more efficient vaccines [99]. In this sense, acellular vaccines provide great advantages, mainly in terms of safety, not only in its production but also in its administration. Nevertheless, the selection of appropriate vaccine candidates requires the study of host-pathogen interaction.

An essential step in establishing a successful infection is the adhesion of microorganisms to eukaryotic cells, resulting in colonization of the tissue involved. Therefore, the molecules involved in this initial interaction have been widely studied as targets for the development of vaccines against various pathogens such as *E. coli* [100,101], *Haemophilus ducreyi* [102], and *Neisseria meningitidis* [103,104] among others. Despite the extensive knowledge of adhesins’ role in the pathogenicity of various bacteria, this group of proteins was not studied well in *Brucella* spp. in terms of immunogenicity and its potential as vaccine candidates.

Al-Mariri et al. studied the immunogenicity and protective efficacy of a DNA vaccine encoding SP41 adhesin from *B. melitensis* in BALB/c mice [105]. Intramuscular (i.m.) administration of pCISP41, a plasmid construct for SP41 expression in mammalian cells, induced SP41-specific serum immunoglobulin G (IgG) antibodies. Moreover, spleen cells from pCISP41-vaccinated mice showed significant T cell proliferation after in vitro stimulation with recombinant SP41 (rSP41) and lysed *B. melitensis*. Splenocytes from pCISP41-immunized animals also responded to rSP41 and bacterial lysate stimulation secreting high levels of gamma interferon (IFN-γ), even though no interleukin-5 (IL-5) was detected. This suggests a predominant T-helper-1 (Th1) immune response. 

After an intraperitoneal challenge with *B. melitensis* 16M, mice immunized with pCISP41 exhibited a reduction of 1.25 and 1.14 log in their spleen burden when compared with control mice at 4-weeks and 8-weeks post-challenge, respectively. Nevertheless, vaccination with attenuated *B. melitensis* Rev-1 strain induced better protection levels than pCISP41 vaccination at both time points, achieving a reduction in spleen burden of 1.79 and 3.17 log, respectively. Although SP41 has been shown to be involved in adhesion to epithelial cells, no studies have been made to evaluate the potential of mucosal or systemic vaccination with this adhesin to protect against *Brucella* infection acquired through mucosae.

*Brucella* infection is frequently acquired through the oral and respiratory routes, and adhesins are anticipated to have a relevant role in these infectious processes. As mentioned above, our results showed that BtaF adhesin from *B. suis* is necessary for complete virulence of *B. suis* after both oral and intratracheal infection [70,71]. In line with these results, we assessed the immunogenic and protective potential of recombinant BtaF when administered intranasally with the mucosal adjuvant Bis-(3’,5’)-cyclic dimeric adenosine monophosphate (c-di-AMP) [71]. To this end, the trimeric form of the BtaF passenger domain fused at the C-terminus to the GCN4tri sequence to facilitate trimerization was successfully expressed and purified [71]. 

BALB/c mice intranasal immunization with BtaF plus c-di-AMP induced high levels of serum BtaF-specific IgG, IgA, IgG1, and IgG2a, and a mixed IgG1/IgG2a profile. In vitro, these serum antibodies reduced *B. suis* infection of human lung epithelial cells (A549 cell line), reduced bacterial binding to fetuin (a protein rich in sialic acid previously described as a ligand for BtaF), and enhanced *B. suis* phagocytosis by murine macrophages. In addition, immunization led to a significant production of specific IgA antibodies in the airways and in the gastrointestinal and genital mucosae.

BtaF immunization induced a systemic and pulmonary Th1 immune response, shown by the secretion of high levels of IFN-γ by splenocytes and lung cells. Furthermore, depletion of CD4+ or CD8+ populations from spleen cells showed that CD4+ cells were responsible for IFN-γ secretion. BtaF-vaccination also triggered the differentiation of specific CD4+ T cells to central memory cells in cervical lymph nodes, and differentiation of T cells to a Th17 profile in the spleen.

Mice vaccination with BtaF plus c-di-AMP demonstrated a high level of protection against *B. suis* oral infection, reducing the splenic burden of *B. suis* by 3.28 log. Unlike the protection achieved against oral infection, intranasal vaccination with BtaF failed to protect against respiratory infection with *B. suis* since no differences were observed in spleen or lung bacterial load between vaccinated and control mice after an intratracheal challenge. 

In summary, despite extensive evidence supporting a role of *Brucella* adhesins in the infectious ability of this pathogen in vivo, only a few studies have assessed the protective efficacy of vaccination with adhesins. Such studies suggest that adhesins hold promise as appropriate antigens for vaccination against oral infection with *Brucella*, even though some protection against systemic infection might be attained. 

## 6. Summary and Future Directions 

For a long time, the process of *Brucella* adhesion to host cells has received little attention. Adhesins of *Brucella* spp. identified to date have been studied with varying degrees of depth. Some of them have been analyzed regarding their roles in the binding to components of the ECM and to different cell types as well as their in vivo role, while, in other cases, their functions have been only evaluated in vitro. Still, for various adhesins, it will be necessary to identify their ligands or cell receptors. The possible use of *Brucella* adhesins as vaccine candidates was tested only in two cases, and one of them showed encouraging results. Therefore, the in vivo role of many of the *Brucella* adhesins identified so far, and their possible applications as a basis for acellular vaccines, remains to be evaluated. It is expected that, in the future, new adhesins will be identified that mediate the initial adhesion of *Brucella* to the remarkable variety of cell types that this pathogen can invade. An interesting perspective will be to characterize the roles of the different adhesin variants from different species/strains and to determine whether they play a role in host preferences.

## Figures and Tables

**Table 1:** Adhesins described in *Brucella* spp.

Adhesin	Organism	KEGG Entry	NCBI Protein ID	Protein Class	Host Ligands Detected	Cellular Adhesion Role	In Vivo Infection Role	Reference
**SP29**	*B. abortus* 9-941	BruAb2_0373	WP_002965789.1	D-ribose ABC transporter substrate-binding protein	Sialic acid-containing proteins	Erythrocytes	ND	[60]
**SP41**	*B. abortus* 9-941	BruAb2_0571	WP_002965982.1	ATP-binding cassette transporter	Sialic acid-containing proteins	Epithelial (HeLa)	No role detected in *B. ovis* infections	[61,62]
**BigA**	*B. abortus* 2308	BAB1_2009	EEP62646.1	Ig-like domain- containing protein	Cell adhesion molecules	Epithelial (HeLa, Caco.2, MDCK)	Potential role in oral infections *	[63,64]
**BigB**	*B. abortus* 2308	BAB1_2012	WP_002967016.1	Ig-like domain- containing protein	Cell adhesion molecules	Epithelial (HeLa)	Potential role in oral infections *	[63,65]
**Bp26**	*B. melitensis 16M*	BMEI0536	WP_002964581.1	Uncharacterized	Type I collagen, vitronectin, fibronectin	ND	ND	[66]
**BmaC**	*B. suis* 1330	BRA1148	WP_006191504.1	Monomeric autotransporter	Fibronectin, type I collagen	Epithelial (HeLa, A549). Synoviocytes. Osteoblasts	ND	[67,68]
**BmaA**	*B. suis* 1330	BR0173	AAN33380.1	Monomeric autotransporter	Fibronectin, type I collagen	Epithelial (HT 29, Caco.2). Synoviocytes. Osteoblasts. Trophoblasts	ND	[68]
**BmaB**	*B. suis* 1330	BR2013	AAN30903.1	Monomeric autotransporter	Fibronectin	Synoviocytes. Osteoblasts. Trophoblasts	ND	[68]
**BtaE**	*B. suis* 1330	BR0072	WP_006191142.1	Trimeric autotransporter	Fibronectin, hyaluronic acid	Epithelial (HeLa, A549)	Mutants display decreased colonization after oral infection	[69]
**BtaF**	*B. suis* 1330	BR1846	A0A0H3G4K1.1	Trimeric autotransporter	Fibronectin, hyaluronic acid, fetuin, type I collagen	Epithelial (HeLa, A549)	Mutants display decreased colonization after oral or respiratory infection	[70,71]

ND: not determined. (*) A mutant lacking the Bab1-2009-2012 genomic island is attenuated in oral infections in mice.
